# Inferior Lateral Genicular Artery Injury during Anterior Cruciate Ligament Reconstruction Surgery

**DOI:** 10.1155/2012/457198

**Published:** 2012-08-26

**Authors:** J. M. Lamo-Espinosa, R. Llombart Blanco, J. R. Valentí

**Affiliations:** Department of Orthopedic and Traumatology Surgery, Clínica Universidad de Navarra, Avenida Pío XII 36, Navarra, 31008 Pamplona, Spain

## Abstract

We report a case of inferior lateral genicular artery (ILG) injury during anterior cruciate ligament (ACL) reconstruction surgery with lateral partial meniscectomy. This is a rare arthroscopy complication. A review of the literature has been made with the aim to define the anatomy of ILG across the lateral articular line and the risk of lesion during knee arthroscopy. We propose embolization as a good treatment option for this type of injuries.

## 1. Introduction

Knee arthroscopy has become a common procedure in clinical practice. The number of vascular complications, still a low percentage, increases in absolute numbers as the technique generalizes.

The global complication rate in knee arthroscopy has been estimated from 0.56% to 8.2% in previous studies. Vascular injuries have been considered rare in all of them [1–4]. The Committee on Complications of the Arthroscopy Association of North America, study in 1986 [2], revealed 12 cases of vascular lesions in a total of 395,566 surgeries, which is 0.003% of them. In long series of experienced arthroscopic surgeons, few vascular injuries are reported. Vincent and Stanish [5] referred only 2 cases of genicular artery (inferior lateral genicular (ILG) and descending genicular) in 2800 procedures.

In literature cases of popliteal artery injury [6, 7], inferior lateral geniculated artery [5, 8–11], inferior medial genicular [11, 12], descending genicular artery [5], as well as branches of the vastus medialis, recurrent anterior tibialis [8], or superior medial genicular artery during knee arthroscopy are found.

We report a case of inferior lateral genicular artery, which occurred during an ACL reconstruction surgery with a bone-tendon-bone (BTB) allograft and single-tunnel technique. A partial meniscectomy was also completed to repair a complex rupture in the anterior horn of the external meniscus. Cases of vascular injuries around the knee published to date refer to open procedures [13] diagnostic arthroscopies [8], arthroscopic meniscectomies [5, 9, 14], and meniscal sutures. These types of lesions had not previously been reported in ACL reconstruction and partial meniscectomy surgeries.

## 2. Case Report 

A 27-year-old male complained of a two-months history of pain and instability in his right knee after a forced varus movement while playing basketball with snapping sensation at the time of injury. He had taken NSAIDs and used a knee brace with partial improvement. He had no comorbidities, and unique remarkable personal antecedent was a previous surgery in that knee because of a patellar tendinopathy.

**Figure 1:**
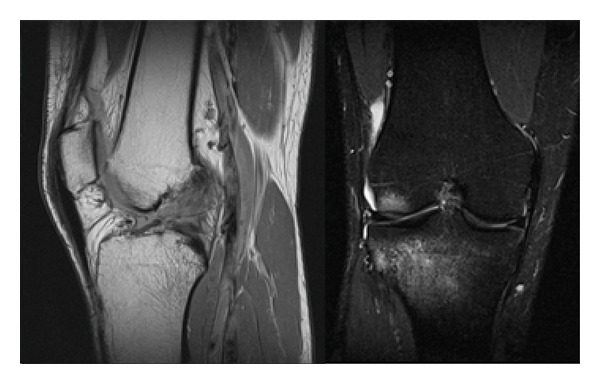
Sagittal T1-weighted MRI shows ACL lesion. Coronal T2 weighted shows hyperintense imaging in the external tibial plateau and lateral femoral condyle.

Physical examination showed knee joint hydrops, positive torn ACL maneuvers (Lachman, pivot-shift, and anterior drawer). Cabot, McMurray, and Graham-Appley tests were positive for lateral meniscus. The KT-1000 test showed a difference of 2 mm compared to the left knee. MRI study revealed the presence of a torn ACL with bone edema in the external tibial plateau and lateral femoral condyle (Figure 1).

Surgical treatment was carried out by a skilled surgeon with more than 4300 arthroscopies of experience. A tourniquet is used with crural ischemia at 300 mmHg. Through the anterolateral and anteromedial portals diagnostic arthroscopy showed an ACL laxity and a complex rupture of the anterior horn of the lateral meniscus. A partial meniscectomy was done so the lateral meniscus was regularized. A third medial incision is made for ACL reconstruction, which was performed with a BTB allograft following the single-tunnel technique. Nonsteroids drugs, thromboprophylaxis, and antibiotic treatment were given following our clinic protocols.

**Figure 2:**
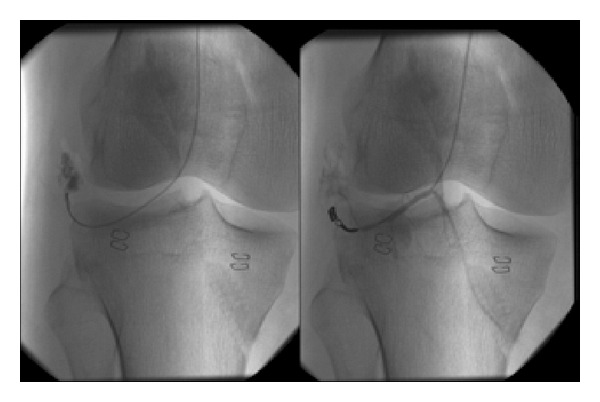
Arteriography imaging after contrast injection, the injury of the inferior lateral genicular (ILG) artery was localized and selectively embolized with microcoils.

First day after surgery, the patient presented active pulsatile bleeding and knee swelling. Arthrocentesis showed haemarthrosis and 60cc were extracted. Compression measures were applied. Given the persistence of bleeding, patient was referred to endovascular treatment. The interventional radiologist performed a selective arteriography via the left common femoral artery using a Judkins 4 French catheter. After contrast injection, the injury of the inferior lateral genicular (ILG) artery was localized and selectively embolized with two microcoils VortX Pushable coil (3 mm × 2.5 mm and 6 mm × 6.5 mm) (Figure 2).

Patient was discharged the day after embolization. He was asymptomatic, and started rehabilitation following the normal protocol for patients after ACL reconstruction.

## 3. Discussion

During ACL reconstruction surgery the injury of inferior medial geniculate (IMG) artery is understandable [12] as the tibial tunnel is placed in the course of the IMG artery area. This makes it harder to explain an injury of the inferolateral branch.

First reference of an inferolateral genicular artery lesion was published by Fairbank and Jamieson in 1951 [13]. They reported two cases of patients who underwent open meniscectomy with submeniscus arthrotomy. Consistent with the idea of Fairbank and Jamieson, Chen et al. [15] studied 11 corpses in order to identify the risk of ILG artery lesion in different meniscal suturing techniques. In his article, he calls the attention on the position of the artery along the external joint line and how on the front the artery crosses the Hoffa fat extrasynovially up to the margin of the external meniscus [5, 8, 10, 15] being at risk when passing needles for suture, or exposing the lateral side of the knee.

Interestingly, damage to the inferolateral genicular artery after arthroscopic procedure was first described in 1987 by Manning and Marshall, in a diagnostic arthroscopic procedure in a patient with ACL rupture [8]. No needles were passed through the joint line. After him others have shown cases of ILG artery injury after arthroscopic meniscectomy [5, 9, 14] and even diagnostic procedures [8, 10].

Supported by the previously reported anatomic basis, authors explain the ILG artery injury after arthroscopy without meniscal suturing because of the placement of the anterolateral portal. Anteromedial and anterolateral portals are carried out systematically in knee arthroscopic procedures. The anterolateral portal is located in close anatomical relationship with the ILG, and may be injured in this surgical gesture.

Unlike previously reported cases, we attribute the damage to the ILG artery in our case to the anterior horn partial meniscectomy of the lateral meniscus. This required the passage of the shaver in the proximity of the synovial capsule. In the picture (Figure 2) the area of the injury does not correspond to the lateral portal, but it does with the area of the meniscal resection. The anterolateral portal is placed medial to the artery damage as shown in the picture. 

Clinical incidence of bleeding in patients undergoing arthroscopic surgery is not high. The branch of the ILG artery has a few millimeters gauge, being considered as a small caliber vessel. Instead of a partial lesion, the damage of the artery will cause a complete section of it, leading to a complete reflect retraction of the vessel. That supposes in most cases a small amount of bleeding [8]. When vessels are not completely damaged, pseudoaneurysms are more likely to form.

In many previously reported cases, vascular injury was unnoticed during the first days or weeks [8, 16]. Delayed diagnosis of vascular damage is marked by ischemia time. The compressive bandage placed before the end of ischemic time during surgery, or the improper use of Doppler may show a good signal in distal vessels [17] with palpable pulses. Also the low incidence of these injuries, make them little suspected by the surgeon.

Gold standard in the treatment of small vessels injuries has been to locate the bleeding point, increase the incision and tie the vessel and resection of the aneurism when present [5, 8, 13, 16]. Nowadays embolization has been used successfully in many cases of this type of injury [14, 18, 19]. Postulated as advantages of this technique are unneeded general anesthesia, minimal exposure with decreases the risk of infection. For larger vessels injuries, such as the popliteal artery, other techniques are recommended. Techniques as reconstruction with or without saphenous vein graft, or angioplasty patching. Angiography, done by a skilled interventional radiologist, has an added value, as it combines diagnosis and treatment in one single procedure.

In conclusion knee surgeons should know the vascular anatomy of the knee, taking into account possible variations of it after previous surgeries. The ILG artery injury should be suspected in cases of bleeding after surgeries including an anterior horn meniscectomy of the external meniscus. Placement of the anterolateral portal can also lead to this artery branch injury. Angiography with selective embolization has become a minimal invasive, good solution for small vessels injuries.
